# A Randomized Controlled Trial of Early versus Late Surgical Decompression for Thoracic and Thoracolumbar Spinal Cord Injury in 73 Patients

**DOI:** 10.1089/neur.2020.0027

**Published:** 2020-09-18

**Authors:** Ali Haghnegahdar, Reza Behjat, Soheil Saadat, Jetan Badhiwala, Majid Reza Farrokhi, Amin Niakan, Keyvan Eghbal, Ehsan Barzideh, Abtin Shahlaee, Fariborz Ghaffarpasand, Zahra Ghodsi, Alexander R. Vaccaro, Mohsen Sadeghi-Naini, Michael G. Fehlings, James David Guest, Pegah Derakhshan, Vafa Rahimi-Movaghar

**Affiliations:** ^1^ Department of Neurosurgery, Shiraz University of Medical Sciences, Trauma Research Center, Shahid Rajaee (Emtiaz) Trauma Hospital, Shiraz, Iran.; ^2^ Department of Emergency Medicine, University of California, Irvine, Irvine, California, USA.; ^3^ Division of Neurosurgery, Department of Surgery, University of Toronto, Toronto, Ontario, Canada.; ^4^ Division of Neurosurgery, Krembil Brain Institute, Toronto Western Hospital, Toronto, Ontario, Canada.; ^5^ Sina Trauma and Surgery Research Center, Tehran University of Medical Sciences, Tehran, Iran.; ^6^ Department of Ophthalmology, University of California, San Francisco, San Francisco, California, USA.; ^7^ Department of Orthopedics and Neurosurgery, Thomas Jefferson University and the Rothman Institute, Philadelphia, Pennsylvania, USA.; ^8^ Department of Neurosurgery, Imam Hossein Hospital, Shahid Beheshti University of Medical Science, Tehran, Iran.; ^9^ Department of Neurosurgery, The Miami Project to Cure Paralysis, University of Miami, Miami, Florida, USA.; ^10^ Brain and Spinal Cord Injury Research Center, Neuroscience Institute, Tehran University of Medical Sciences, Tehran, Iran.; ^11^ Department of Neurosurgery, Shariati Hospital, Tehran University of Medical Sciences, Tehran, Iran.; ^12^ Pre-Hospital and Hospital Emergency Research Center, Tehran University of Medical Sciences, Tehran, Iran.; ^13^ Tissue Repair Laboratory, Institute of Biochemistry and Biophysics (IBB), University of Tehran, Tehran, Iran.; ^14^ Shiraz Neuroscience Research Center, Shiraz University of Medical Sciences, Shiraz, Iran.; ^15^ Department of Neurosurgery, Shiraz University of Medical Sciences, Shiraz, Iran.

**Keywords:** acute, lumbar cord, randomized controlled trial, spinal cord injuries, thoracic cord, traumatic

## Abstract

Convincing clinical evidence exists to support early surgical decompression in the setting of cervical spinal cord injury (SCI). However, clinical evidence on the effect of early surgery in patients with thoracic and thoracolumbar (from T1 to L1 [T1–L1]) SCI is lacking and a critical knowledge gap remains. This randomized controlled trial (RCT) sought to evaluate the safety and efficacy of early (<24 h) compared with late (24–72 h) decompressive surgery after T1–L1 SCI. From 2010 to 2018, patients (≥16 years of age) with acute T1–L1 SCI presenting to a single trauma center were randomized to receive either early (<24 h) or late (24–72 h) surgical decompression. The primary outcome was an ordinal change in American Spinal Injury Association (ASIA) Impairment Scale (AIS) grade at 12-month follow-up. Secondary outcomes included complications and change in ASIA motor score (AMS) at 12 months. Outcome assessors were blinded to treatment assignment.

Of 73 individuals whose treatment followed the study protocol, 37 received early surgery and 36 underwent late surgery. The mean age was 29.74 ± 11.4 years. In the early group 45.9% of patients and in the late group 33.3% of patients had a ≥1-grade improvement in AIS (odds ratio [OR] 1.70, 95% confidence interval [CI]: 0.66-4.39, *p* = 0.271); significantly more patients in the early (24.3%) than late (5.6%) surgery group had a ≥2-grade improvement in AIS (OR 5.46, 95% CI: 1.09-27.38, *p* = 0.025). There was no statistically significant difference in the secondary outcome measures. Surgical decompression within 24 h of acute traumatic T1–L1 SCI is safe and is associated with improved neurological outcome, defined as at least a 2-grade improvement in AIS at 12 months.

## Introduction

Acute traumatic spinal cord injury (SCI) is a devastating disorder that exerts a significant physical, emotional, and economic burden on patients, families, and society at large.^1,2^ The pathophysiology of SCI involves primary and secondary injury mechanisms; the rationale for expeditious surgical decompression in the setting of acute SCI is that it may mitigate secondary injury, improving long-term neurological outcomes.^3,4^ To this end, pre-clinical studies have demonstrated better neurobehavioral outcomes in rodent models of SCI when the time from injury to decompression is minimized.^5–9^ There are few randomized human studies evaluating the effect of early surgical decompression for acute SCI.^10^ Currently, the highest quality evidence comes from the Surgical Timing in Acute Spinal Cord Injury Study (STASCIS), which demonstrated that early (<24 h) surgical decompression was associated with greater odds of achieving a 2-or-more grade improvement in the American Spinal Injury Association (ASIA) Impairment Scale (AIS), as compared with late (≥24 h) surgery, in patients with acute cervical SCI.^11^ On the basis of this and other studies, clinical practice guidelines developed and published by AO Spine provide a weak recommendation based on low-quality evidence that “early surgery be offered as an option for acute SCI patients regardless of level.”^12^

Although STASCIS addressed the question of early surgery for cervical SCI, a critical knowledge gap remains on the efficacy of early decompressive surgery in the setting of thoracic and thoracolumbar SCI. Thoracic and thoracolumbar SCIs have distinct biomechanical and physiological characteristics; most notably, these injuries often result from high-energy trauma and are more likely to result in complete injury compared with other regions,^13^ and the tenuous vascular supply to the region may lead to reduced potential for recovery.^14,15^ The natural history of thoracic SCI has been thought to involve minimal recovery, which occurs significantly more in low-thoracic than high/mid-thoracic SCIs.^16^ Improved conversion rates were observed in all registries for low-thoracic (T10–T12) injuries when compared with high/mid-thoracic (T2–T9) injuries. Given the paucity of evidence in this domain, the present study sought to compare the neurological outcomes of early (<24 h) versus late (24–72 h) surgical decompression in patients with thoracic and thoracolumbar SCI.

## Methods

### Trial design and oversight

In this randomized controlled trial (RCT), conducted from September 2010 to April 2018, patients with thoracic and thoracolumbar SCI presenting to the Neurosurgery Department of Shahid Rajaee Hospital, Shiraz University of Medical Sciences were randomized in a 1:1 ratio to receive early (<24 h; intervention) or late (24–72 h; control) surgical decompression. We generated the randomization during the protocol preparation but when logistic and clinical problems precluded us from commitment to the randomization sequence, some patients crossed over to the other group. Crossovers at the allocation phase are described in the Results section. Randomization was by permuted blocks stratified by complete (AIS grade A) versus incomplete (AIS grades B, C, D) SCI. The randomization scheme was generated centrally by a scientist who was not involved in determining study eligibility. We generated the randomization using sealedenvelope.com when preparing the protocol, and the randomization of study subjects was concealed in sealed, opaque, sequentially numbered envelopes. By nature, subjects and physicians could not be blinded to treatment. However, outcome residents (senior neurosurgery resident) trained to undertake the examinations in the RCT remained blinded to treatment group allocation throughout randomization and follow-up.

The trial was registered with the ISRCTN Registry (ISRCTN61263382). The study protocol has been published in the peer-reviewed literature previously.^17^ Randomization and data collection, analysis, and interpretation were conducted at Sina Trauma and Surgery Research Center of Tehran University of Medical Sciences. This study was approved by the Research Ethics Board of the Sina Trauma and Surgery Research Center of Tehran University Medical Sciences. Written informed consent was obtained from all participants prior to enrollment.

There are approximately 30–40 cases of thoracolumbar SCI seen at Shahid Rajaee Hospital per year, but only about 25% of these (6–10 case per year) present within 24 h of injury. A primary report of the study was published with 35 cases.^18^ Based on *a priori* calculations, the target sample size was 328 patients.^17^ However, the trial had to be terminated early, at 73 patients, due to slower than anticipated patient recruitment, which is a well-recognized challenge in conducting clinical trials for SCI.^19,20^ This logistic problem prevented us from continuing the study. After 10 years, the local principal investigator moved to a different country and we lost direct supervision of the project and residents, and so we stopped the study to prevent a decrease in study quality.

### Participants

Patients (≥ 16 years of age) with acute traumatic thoracic and thoracolumbar SCI (T1–L1) and who were hemodynamically stable, had evidence of spinal cord compression on magnetic resonance imaging (MRI), and presented less than 24 h of injury were eligible. Initial patient assessment was performed twice. The first clinical examination was by a junior resident at admission and the second was by a senior resident or local principal investigator at least 1 h prior to the surgery, who confirmed the previous examination. Detailed inclusion and exclusion criteria are presented in Table 1. Clinical parameters collected at baseline included age, sex, level of injury, mechanism of injury, and time from injury to admission.

**Table 1.** Eligibility Criteria of Early vs. Late Surgical Decompression for Acute Traumatic Thoracic and Thoracolumbar Spinal Cord Injury

Inclusion criteria	Exclusion criteria
Age ≥16 years	Concomitant traumatic brain injury^a^
Acute traumatic SCI with neurological level from T1 to L1	Pre-injury major medical comorbidity^b^
Hemodynamic stability	Pre-injury major neurological deficits or disease^c^
Spinal cord compression confirmed by MRI	Current major psychiatric illness
Hospital admission within 24 h of injury	Ankylosing spondylitis
Patient or proxy able and willing to provide informed consent	Penetrating spinal injury
	Life-threatening injuries that prevent early spinal cord decompression
	Pregnancy
	Criminals under incarceration
	Spinal shock^d^
	Cognitive impairment preventing accurate neurological assessment
	Injury involving more than two adjacent vertebral levels

^a^ Defined as an altered level of consciousness (GCS score ≤14)

^b^ Includes myocardial infarction within 3 months, uncompensated congestive heart failure, active systemic malignancy, AIDS, and diabetes mellitus.

^c^ Includes hemiparesis, paraparesis, or quadriparesis; stroke; Parkinson's disease; syringomyelia; and Guillain-Barré syndrome.

^d^ Defined as areflexia and autonomic dysfunction.

GCS, Glasgow Coma Scale; MRI, magnetic resonance imaging; SCI, spinal cord injury.

### Interventions

All surgeries were performed at a single trauma center, Shiraz University of Medical Sciences. Standard spinal immobilization techniques were used. Patients were resuscitated in accordance with the standard Advanced Trauma Life Support (ATLS) protocol. We infused methylprednisolone sodium succinate for all eligible patients admitted within 24 h of injury. The dose of intravenous methylprednisolone sodium succinate was a 30 mg/kg bolus and a 5.4 mg/kg/h infusion for 24 h. Patients underwent long- or short-segment posterior fixation with or without instrumentation of the fractured vertebrae, or 360-degree fixation, at the discretion of the treating surgeon.

### Outcomes

Neurological outcomes were evaluated by standard ASIA/International Standards for Neurological Classification of Spinal Cord Injury (ISNCSCI)^21^ examination at enrollment and 1, 3, 6, and 12 months post-operatively by trial coordinators who were unaware of treatment assignment. The primary end-point was an ordinal change in AIS grade at 12-month follow-up. Change in ASIA motor score (AMS) at 12 months was evaluated as a secondary end-point.

#### Safety outcomes

Major and minor adverse events, including mortality, were recorded on an ongoing basis throughout the study period, from screening to end of study participation.

### Statistical analysis

All analyses followed the intention-to-treat principle. Descriptive statistics were by the mean and standard deviation for continuous variables and count and percentage for categorical variables. Between-group comparisons of ≥1-grade and ≥2-grade improvements in AIS were made by chi-square test. The change in AMS from baseline to 12 months in the two groups was compared with use of a *t* test. All statistical analyses were performed using Stata 15 (Stata Corp., College Station, TX, USA) with an *a priori* specified significance level of *p* = 0.05 (two-tailed).

## Results

### Patients

**FIG. 1.**
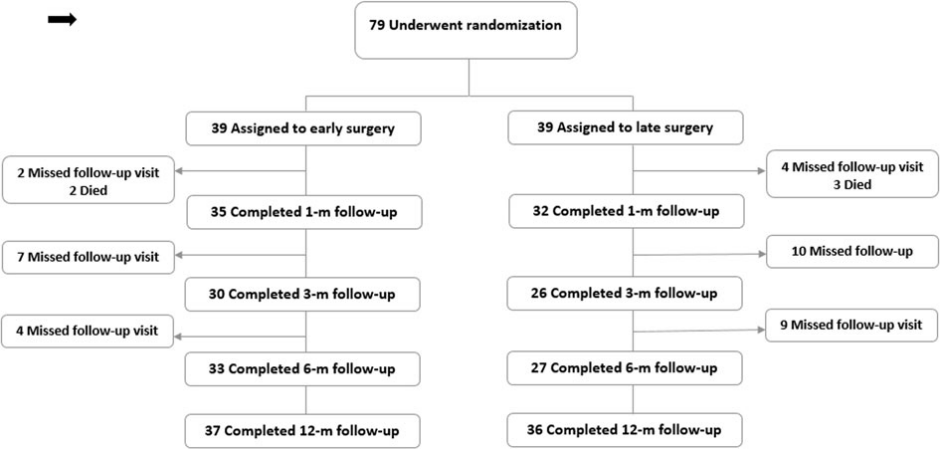
Flowchart of patient eligibility, randomization, and follow-up.

**FIG. 2.**
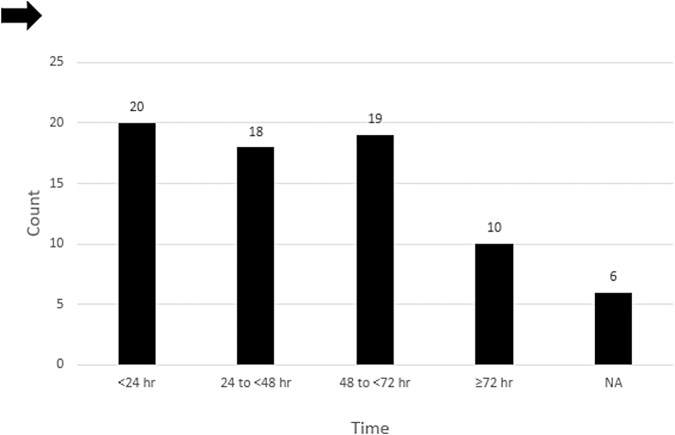
Surgical timing in both the early and late groups (*n* = 73).

Patient enrollment, randomization, and follow-up are summarized in Figure 1. A total of 78 participants underwent randomization, of which 39 underwent early and 39 late surgical decompression. Five patients (6.4%) died during acute hospitalization (*n* = 2, early surgery; *n* = 3, late surgery) and were excluded from further analysis. The remaining 73 patients (*n* = 37, early surgery; *n* = 36, late surgery) formed the study cohort. From the early group of 37 patients, 15 underwent surgery in less than 24 h, and 17 patients crossed over to the late surgery group. The crossover patients were analyzed based on the intention-to-treat (ITT) assignment (Fig. 2). The exact times lapsed for 5 patients allocated to the early surgery group were missed. In the late group, 30 patients underwent surgery ≥24 h, and 5 patients crossed over to the early surgery group. The exact time lapsed for 1 patient allocated in the late surgery group was missed. Baseline patient and injury characteristics (Table 2) were balanced between treatment groups. The mean age of the overall study cohort was 29.74 ± 11.4 years. There were 19 females (26.0%). The most common injury mechanism was a motor vehicle collision (41; 56.2%). The majority of patients (41; 56.2%) had a complete injury (AIS grade A). Mean AMS was 60.2.

**Table 2.** Baseline Characteristics of Early vs. Late Surgical Decompression for Traumatic Thoracic and Thoracolumbar Spinal Cord Injury (*n* = 73), *n* (%)

Characteristic	Early surgery,* n* = 37	Late surgery,* n* = 36	P-value
**Age, mean ± SD**	29.7 ± 10.3	34.9 ± 12.0	0.057
**Female sex, no. (%)**	9 (24.3)	10 (27.8)	0.79
**Mechanism of injury**			
**Motor vehicle collision or crashes**	17 (45.9)	24 (66.7)	0.09
**Falls**	18 (48.7)	11 (30.6)	
**Sport**	0 (0.0)	0 (0.0)	
**Other**	2 (5.4)	0 (0.0)	
**Missing**	0 (0.0)	1 (2.8)	
**AIS grade, no. (%)**			1.00
**A**	21 (56.8)	20 (55.6)	
**B**	5 (13.5)	5 (13.9)	
**C**	4 (10.8)	4 (11.1)	
**D**	7 (18.9)	7 (19.4)	
**Neurological level number (%)**			0.25
**T1-4**	1 (2.7)	4 (11.1)	
**T5-8**	5 (13.5)	7 (19.4)	
**T9-L1**	31 (83.8)	25 (69.4)	
**AMS, mean ± SD**	62.3 ± 15.6	58.1 ± 14.1	0.23
**Fracture morphology ^a^**			0.71
**Type A**	17 (45.9)	13 (36.1)	
**Type B**	7 (18.9)	9 (25.0)	
**Type C**	12 (32.4)	14 (38.9)	

^a^ Based on the AO Spine Thoracolumbar Spine Injury Classification System.^21^

AIS, American Spinal Injury Association (ASIA) Impairment Scale; AMS, ASIA motor score; SD, standard deviation.

We provide the details of patients' data in Supplementary Table S1. 

### Primary outcome measure

**FIG. 3.**
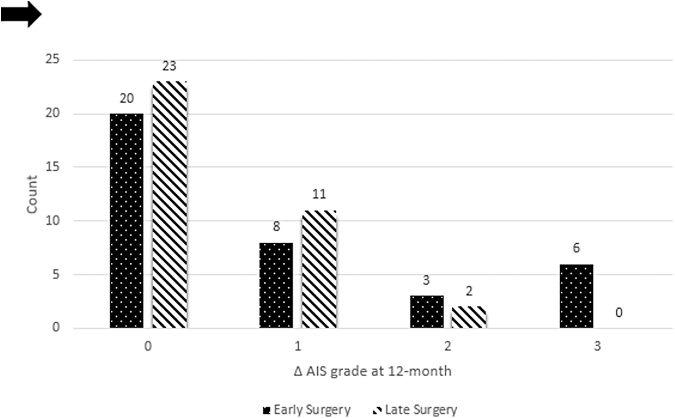
American Spinal Injury Association (ASIA) Impairment Scale (AIS) grade improvement at 12-month follow-up with early versus late surgery.

In total, 29 patients (39.7%) experienced an improvement in AIS grade at 12 months. In the early surgery group, AIS grade improvement at 12 months was as follows: 20 patients (54.1%) had no improvement, 8 (21.6%) had a 1-grade improvement, 3 (8.1%) had a 2-grade improvement, and 6 (16.2%) had a 3-grade improvement. Five out of 21 (23.8%) of AIS A patients had a 3-grade improvement (Table 3). In the late surgery group, improvement in AIS grade at 12 months was as follows: 24 patients (66.7%) had no improvement, 10 (27.8%) had a 1-grade improvement, 2 (5.6%) had a 2-grade improvement, and no patients had a 3-grade improvement (Table 4). Accordingly, 17 patients (45.9%) in the early surgery group and 12 (33.3%) in the late surgery group had a ≥1-grade improvement in AIS at 12 months (early vs. late; OR 1.70, 95% CI: 0.66-4.39, *p* = 0.271), and 9 patients (24.3%) in the early surgery group and 2 (5.6%) in the late surgery group had a ≥2-grade improvement in AIS at 12 months (early vs. late; OR 5.46, 95% CI: 1.09-27.38, *p* = 0.025; Table 5 and Fig. 3).

**Table 3.** Ordinal Change in AIS grade from Baseline to 12-month Follow-Up: Early surgery group (*n* = 37)

		12-month AIS grade					
		A	B	C	D	E	Total
*Pre-operative AIS grade*	*A*	16	0	0	5	0	21
	*B*	0	0	3	1	1	5
	*C*	0	0	0	2	2	4
	*D*	0	0	0	4	3	5

AIS, American Spinal Injury Association (ASIA) Impairment Scale.

**Table 4.** Ordinal Change in AIS Grade from Baseline to 12-Month Follow-Up: Late Surgery Group (*n* = 36)

		12-month AIS grade					
		A	B	C	D	E	Total
*Pre-operative AIS grade*	*A*	19	0	1	0	0	20
	*B*	0	0	4	1	0	5
	*C*	0	0	0	4	0	4
	*D*	0	0	0	5	2	7

AIS, American Spinal Injury Association (ASIA) Impairment Scale.

**Table 5.** Comparison of AIS Grade Conversions in Early vs. Late Surgery Groups

Outcome	Early surgery,* n* = 37	Late surgery,* n* = 36	OR (95% CI)	P-value
≥1-grade AIS improvement	17 (45.9)	12 (33.3)	1.70 (0.66-4.39)	0.27
≥2-grade AIS improvement	9 (24.3)	2 (5.6)	5.46 (1.09-27.38)	0.037^*^

^*^ Statistically significant (*p* < 0.05).

AIS, American Spinal Injury Association (ASIA) Impairment Scale; CI, confidence interval; OR, odds ratio.

### Secondary outcome measures and adverse events

Mean AMS at 12 months was 71.3 ± 20.5 points (mean 75.1 ± 21.2 points, early surgery; mean 67.3 ± 19.2 points, late surgery). In the overall study cohort, AMS improved by a mean of 11.1 ± 11.7 points at 12 months. Improvements in AMS at 12 months were comparable between early (mean 12.8, 95% CI: 8.6-17.1 points) and late (mean 9.2 points, 95% CI: 5.7-12.7 points) surgery groups (*p* = 0.235). Patients with incomplete SCI experienced greater improvement in AMS than patients with complete SCI, in both early and late decompression groups (*p* < 0.001).

Three patients in the early surgery group developed a deep vein thrombosis and three laterally placed screws were revised the same day of surgery. Eleven patients in the late surgery group experienced complications, including deep vein thrombosis (*n* = 2), laterally placed screws (*n* = 3 screws in two cases), bilateral rod fracture (*n* = 1), a delayed pulled-out screw (*n* = 1), wound infection (*n* = 2), cerebrospinal fluid (CSF) leak (*n* = 1), meningitis (*n* = 1), and decubitus ulcer (*n* = 1). According to our study design, there was no complication reported related to the methylprednisolone therapy.

## Discussion

The present study is a prospective RCT comparing early versus late surgery in acute traumatic thoracic and thoracolumbar SCI. The primary analysis indicates a significant difference, in favor of early surgery versus late surgery, for a >2-grade AIS conversion at 12-month follow-up. A 2-grade AIS change was considered clinically significant in the Sygen multi-center acute spinal cord injury study.^22^ Improvements in AMS at 12 months were comparable between early and late surgery groups.

A Chinese single-center prospective cohort study of 721 patients with thoracic and thoracolumbar incomplete SCI also divided patients into an early (<24 h after SCI, *n* = 335) and a late (24–72 h after SCI, *n* = 386) decompression group. Each group was divided into A, B, and C subgroups according to the AO Spine Injury Classification System (AOSICS). Analysis of the effect of surgical timing on the AIS grade conversion showed that early surgical decompression did not lead to significant AIS improvement in AOSICS classification type A, but there was improved conversion in AOSICS classification type B and type C. The authors concluded that patients classified with AOSICS type A with complete SCI do not have to undergo aggressive early operations. However, patients with type B and type C injuries should undergo an early operation to achieve better clinical results.^23^

Yousefifard and colleagues in a systematic review and meta-analysis indicated that surgical decompression within 24 h can improve neurological recovery by 0.75 (95% CI: 0.63-0.90; *p* = 0.002).^24^ The pooled recovery rate (RR) was 0.77 (95% CI: 0.68-0.89) for at least 1-grade neurological improvement, and 0.84 (95% CI: 0.77-0.92) for at least 2-grade improvement. Further, surgical decompression performed within 24 h after injury was found to be associated with significantly lower rates of post-surgical complications (RR = 0.77; 95% CI: 0.68-0.86; *p* < 0.001). In the current RCT, we did not find a significantly lower complication rate, perhaps due to low sample size.

Khorasanizadeh and associates in a recent meta-analysis on thoracic SCI^13^ evaluated 114 studies for AIS/Frankel changes in 19,913 patients and AMS changes in 6920 patients. The overall AIS conversion rate was 19.3% (95% CI: 16.2-22.6) for patients with grade A. Considering baseline neurological impairment as the most important prognostic factor for neurological recovery, patients with incomplete SCI experienced more significant improvement in AMS than patients with complete SCI in both early and late decompression groups. This is in line with previous findings, which suggest that patients with complete SCI have poor neurological recovery. In the above-mentioned meta-analysis, the lowest neurological RR was significantly different between all grades of SCI severity and the lowest RR was in AIS A in the following order: C > B > D > A. The lowest RR according to the level of injury was thoracic, which was in the following pattern: lumbar > cervical and thoracolumbar > thoracic.

Khorasanizadeh and associates have shown that studies with follow-up durations of approximately 6 months or less reported significantly lower RRs for incomplete SCI compared with studies with long-term follow-ups.^13^ We followed the patients in our study for at least a year. Interestingly, 5 patients (out of 19 patients) with AIS grade A in the early surgery group improved to AIS grade D at 1-year follow-up, but only 1 patient (out of 19 patients) in the late surgery group with AIS grade A improved to grade C in the same time period. Among 6 patients who recovered from complete SCI, the level of injury was T5 (*n* = 1), T11 (*n* = 1), and T12 (*n* = 4). As such, our findings support that early decompression enhances the chance for future improvement especially in the lower thoracic, which 83.3% of improvement in our study belongs to the lower thoracic.

Bourassa-Moreau and coworkers suggested that early surgical decompression improved the AIS score more than late surgical decompression in patients with complete cervical SCI, but not in those with thoracic and thoracolumbar SCI.^25^ El Tecle and colleagues in a systematic review of 11 RCTs and 9 observational studies with 1162 patients with AIS A reported that patients who underwent early surgery had a higher rate of conversion (46.1%) than patients who underwent late surgery (25%; OR 2.31, 95% CI: 1.08-4.96, *p* = 0.03).^26^ In a retrospective cohort study in 86 patients with traumatic SCI in all levels, Kim and associates showed that AIS grade improvement was significantly greater in the early (<48 h) than in the late group (>48 h; *p* = 0.039). AIS grade improvement was also significantly greater in the incomplete SCI group than in the complete SCI group.^27^

According to an updated literature review using the GRADE approach, a clinical guideline with low-quality evidence suggests that “early surgery can be offered as an option for acute SCI patients regardless of level.”^12^ But due to a lack of high-quality evidence, the challenge of the surgical timing remains unsolved. A recent meta-analysis based on observational studies in 948 patients with thoracic SCI showed there was no superiority of either early or late surgical decompression in neurological RR.^28^

Another meta-analysis indicates motor RR after traumatic SCI depends on injury factors (i.e., injury severity, level, and mechanism of injury). Interestingly, type of treatment (surgical decompression vs. conservative treatment) and country of origin (developing vs. developed countries) do not have significant effect on the motor RR.^13^ In a meta-analysis from 1683 patients (990 with incomplete neurological deficit), patients with incomplete SCI who underwent early (<24 h) surgery had greater neurological improvement than late surgical decompression and conservative management alone. However, there was only one prospective randomized study and eight prospective non-randomized case series.^29^ In another systematic review of two prospective and eight retrospective observational studies with 1427 patients with thoracolumbar traumatic SCI, early surgery was associated with fewer complications, shorter hospital stay, and shorter intensive care unit (ICU) stay. However, the effect of early decompression on the neurological outcome remains unclear.^30^

Based on previous feasibility studies, due to transport and lifesaving protocols, only 23.5–51.4% of patients with SCI can undergo an operation within the first 24 h after injury.^31^ This rate may also be reduced in thoracic SCI due to high-energy multiple trauma that is needed to produce thoracic SCI, which produces more injuries that can complicate early surgery. We, therefore, excluded patients with life-threatening injuries that prevent early spinal cord decompression.

In the current study, all patients regardless of their treatment groups underwent assignment of AIS grade within the first hour after emergency admission. Burns and colleagues showed that patients who are classified as AIS A within 72 h post-injury remain grade A in 80% of cases, with about 10% converting to AIS B and about 10% converting to AIS C.^32^ Scivoletto and associates showed that, if the baseline assessment is performed after 72 h post-injury, the percentage of improvement decreases dramatically, to 2.5%.^33^ The most important answer to this significant criticism of the study protocol is randomization.

To ascertain the validity of the AIS grade we also excluded patients with moderate traumatic brain injury (Glasgow Coma Scale [GCS] score ≤14) or patients with life-threatening injury. Another condition that could violate the validity of the AIS grade assessed in the emergency department is spinal shock. However, spinal shock is a dynamic physiological continuum consisting of four phases, in all patients with SCI beginning from the injury up to a year.^34^ Therefore, it would be inappropriate to classify patients into two groups; with and without spinal shock. In addition, in the current study there were trends toward potential differences between the two groups with regard to age and cause of injury. However, Table 2 presents the ITT population, and therefore these differences could not have arisen due to crossovers. Rather, given the nature of randomization, these baseline differences arose by chance alone, as is often seen in RCTs. In our protocol and study design, defined *a priori*, there was no specific plan to adjust for baseline covariates. Therefore, we believe it would be a violation of the protocol to now adjust for these baseline characteristics that were found to be different. Nonetheless, given the small sample size, we do agree that these could influence the results to some degree.

### Limitations

The present study has some limitations. First, due to problems in the hospital setting, we could not evaluate all outcomes of interest predefined in the protocol. Therefore, we focused on the AIS conversion rate, AMS, and complications during the hospital admission. Also, repeated evaluations of AIS and AMS in serial follow-up, as mentioned in the protocol, were missed in some subjects during the executory phase at 1, 3, and 6 months. In addition, especially the 1- and 3-month follow-up evaluation and comparison were premature and less substantial than the 12-month outcome. In the interpretation of the results, this lapse suggests some degree of attrition bias from 1- to 6-month follow-up. Second, the causes of mortality in five case patients (two in the early decompression group and three in the late decompression group) were not recorded. Further, due to limited available information regarding comorbidities in study patients, we were not able to conduct additional analyses regarding these conditions. Third, after 9 years of patient recruitment, we had recruited 73 patients or just one-fourth of the desired sample size. Because we could not continue the trial due to the logistic problem, the potential for selection bias is high and analysis is underpowered to detect the minimum clinical difference in the AMS between early and late decompression groups. To address this issue, we provide details, including individual patient data (IPD), in Supplementary Table S1, to enable future systematic reviews to perform IPD meta-analysis and to derive meaningful conclusions.

## Conclusion

Surgical decompression within 24 h of acute traumatic thoracic and thoracolumbar SCI is safe and associated with improved neurological outcomes, defined as at least a 2-grade improvement in AIS at 12 months. AIS grade and AMS were improved in both the early and late surgical decompression groups after traumatic SCI. Greater improvement of both AIS ≥1-grade and AMS was observed in the early group, although it was not significant versus the late group. The discrepancy between AIS and AMS might be due to the low sample size/low power of the analysis. Future RCTs with greater sample sizes are needed to confirm these findings.

## Supplementary Material

Supplemental data

## Notes

**Cite this article as:** Haghnegahdar A, Behjat R, Saadat S, Badhiwala J, Farrokhi MR, Niakan A, Eghbal K, Barzideh E, Shahlaee A, Ghaffarpasand F, Ghodsi Z, Vaccaro AR, Sadeghi-Naini M, Fehlings MG, Guest JD, Derakhshan P, Rahimi-Movaghar V (2020) A randomized controlled trial of early versus late surgical decompression for thoracic and thoracolumbar spinal cord injury in 73 patients, *Neurotrauma Reports* 1:1, 78–87, DOI:10.1089/neur.2020.00027.

## Abbreviations Used

AIS: American Spinal Injury Association Impairment Scale
AMS: ASIA motor score
AOSICS: AO Spine Injury Classification System
ASIA: American Spinal Injury Association
ATLS: Advanced Trauma Life Support
CI: confidence interval
CSF: cerebrospinal fluid
GCS: Glasgow Coma Scale
ICU: intensive care unit
IPD: individual patient data
ISNCSCI: International Standards for Neurological Classification of Spinal Cord Injury
ITT: intention-to-treat
MRI: magnetic resonance imaging
OR: odds ratio
RCT: randomized controlled trial
RR: recovery rate
SCI: spinal cord injury
SD: standard deviation
STASCIS: Surgical Timing in Acute Spinal Cord Injury Study
T1–L1: from T1 to L1
