# Supplementary material for: A Randomized Controlled Trial of Early versus Late Surgical Decompression for Thoracic and Thoracolumbar Spinal Cord Injury in 73 Patients
Source: Neurotrauma Rep. 2020 Sep 18;1(1):78–87. doi: 10.1089/neur.2020.0027 (PMC8240887; doi:10.1089/neur.2020.0027)
Supplement: Supplemental data [file Supp_Table1.doc]

Supplementary table 1 – Patient data in detail

| **Patient's number** | **Age** | **Gender** | **A to A (hrs.)** | **Injury mechanism** | **A-OR (hrs.)** | **Group Allocation** | **Adm-AIS** | **Adm-AMS** | **Fracture type** | **Spine level** | **Surgical procedure** | **F/U** | **FU-AMS** | **FU-AIS** | **AIS-C** |
| --- | --- | --- | --- | --- | --- | --- | --- | --- | --- | --- | --- | --- | --- | --- | --- |
| 1 | 31 | M | 6.3 | 2 | 65 | 2 | D | 89 | 2 | L1 | 2 | 12 | 100 | E | 1 |
| 2 | 43 | M | 3 | 2 | 46 | 2 | B | 50 | 3 | T12 | 2 | 12 | 68 | C | 1 |
| 3 | 34 | M | 8.3 | 2 | 22 | 1 | B | 50 | 3 | T12 | 2 | 12 | 78 | C | 1 |
| 4 | 67 | M | 9 | 1 | 31 | 2 | B | 50 | 4 | T11 | 2 | 12 | 77 | D | 2 |
| 5 | 21 | M | 0.5 | 2 | 29 | 2 | B | 50 | 3 | T12 | 2 | 12 | 69 | C | 1 |
| 6 | 22 | M | 12 | 1 | 26 | 1 | A | 50 | 4 | T5 | 2 | 12 | 50 | A | 0 |
| 7 | 24 | M | 0.5 | 2 | 42 | 2 | A | 50 | 4 | T12 | 2 | 12 | 50 | A | 0 |
| 8 | 50 | F | 3.25 | 2 | 46 | 2 | A | 50 | 4 | T12 | 2 | 12 | 50 | A | 0 |
| 9 | 32 | M | 1 | 2 | 39 | 2 | A | 50 | 3 | T6 | 2 | 12 | 50 | A | 0 |
| 10 | 45 | M | 1.5 | 2 | 8.5 | 2 | A | 50 | 3 | T8 | 2 | 12 | 50 | A | 0 |
| 11 | 57 | M | 1 | 2 | 22 | 2 | A | 50 | 3 | T11 | 2 | 12 | 50 | A | 0 |
| 12 | 25 | M | 6.25 | 1 | 48 | 2 | A | 50 | 2 | T12 | 3 | 12 | 50 | A | 0 |
| 13 | 26 | M | 1.75 | 1 | 62 | 2 | B | 50 | 2 | T9 | 2 | 12 | 70 | C | 1 |
| 14 | 22 | M | 4.25 | 2 | 50 | 2 | A | 50 | 4 | T8 | 2 | 12 | 50 | A | 0 |
| 15 | 35 | M | 6 | 4 | 22 | 1 | A | 50 | 4 | T7 | 2 | 12 | 50 | A | 0 |
| 16 | 51 | M | 9 | 2 | 23 | 1 | C | 68 | 3 | L1 | 2 | 12 | 100 | E | 2 |
| 17 | 45 | F | 1.25 | 1 | 34 | 1 | A | 50 | 4 | T9 | 2 | 12 | 50 | A | 0 |
| 18 | 22 | M | 2.25 | 2 | 64 | 1 | C | 58 | 2 | L1 | 3 | 12 | 100 | D** | 1 |
| 19 | 27 | M | 18.75 | 2 | 51 | 2 | D | 84 | 2 | L1 | 3 | 12 | 98 | D | 0 |
| 20 | 52 | F | 1 | 2 | 22.5 | 2 | D | 90 | 2 | T12 | 2 | 12 | 99 | D | 0 |
| 21 | 32 | F | 0.75 | 1 | 20 | 1 | B | 52 | 4 | T12 | 3 | 12 | 72 | C | 1 |
| 22 | 17 | M | 2 | 2 | 11 | 1 | A | 50 | 4 | L1 | 2 | 12 | 50 | A | 0 |
| 23 | 46 | M | 4 | 2 | 30 | 2 | A | 50 | 4 | T12 | 2 | 12 | 50 | A | 0 |
| 24 | 18 | F | 2 | 2 | 18 | 1 | D | 94 | 2 | T12 | 2 | 12 | 100 | E | 1 |
| 25 | 24 | M | 1 | 1 | 17 | 1 | C | 70 | 3 | L1 | 2 | 12 | 77 | D | 1 |
| 26 | 34 | M | 1.25 | 2 | 39 | 2 | B | 50 | 2 | L1 | 2 | 12 | 62 | C | 1 |
| 27 | 27 | F | 5.25 | 2 | 48 | 2 | A | 50 | 4 | T8 | 2 | 12 | 50 | A | 0 |
| 28 | 28 | F | 8.5 | 2 | 19 | 1 | A | 50 | 4 | L1 | 2 | 12 | 50 | A | 0 |
| 29 | 28 | M | 1 | 2 | 20 | 1 | A | 50 | 4 | T11 | 2 | 12 | 50 | A | 0 |
| 30 | 24 | M | 2 | 1 | 42 | 2 | A | 50 | 4 | T12 | NA | 12 | 56 | A | 0 |
| 31 | 34 | F | 16 | 2 | 76 | 2 | A | 50 | 4 | T8 | 3 | 12 | 50 | A | 0 |
| 32 | 36 | M | 6.25 | 2 | 50 | 2 | C | 62 | 4 | T2 | 2 | 12 | 93 | D | 1 |
| 33 | 57 | M | 10.4 | 2 | 19 | 1 | D | 82 | 2 | T12 | 2 | 12 | 98 | D | 0 |
| 34 | 39 | F | 2 | 2 | 55 | 2 | D | 82 | 3 | T12 | 2 | 12 | 99 | D | 0 |
| 35 | 36 | M | 2 | 1 | 46 | 2 | A | 50 | 4 | T12 | 2 | 12 | 50 | A | 0 |
| 36 | 38 | M | 3 | 2 | 12 | 2 | C | 53 | 2 | L1 | 2 | 12 | 99 | D | 1 |
| 37 | NA | M | 1 | 1 | NA | 1 | B | 62 | 2 | T12 | NA | 12 | 95 | D | 2 |
| 38 | 19 | M | 3 | 1 | 30 | 1 | B | 50 | 2 | L1 | 2 | 12 | 100 | E | 3 |
| 39 | 25 | F | 1 | 2 | 8 | 1 | A | 50 | 4 | T10 | 2 | 12 | 50 | A | 0 |
| 40 | 17 | M | 0.5 | 2 | 67 | 2 | A | 50 | 4 | T6 | 2 | 12 | 56 | A | 0 |
| 41 | 38 | F | 1 | 1 | 40 | 2 | D | 76 | 2 | L1 | 2 | 12 | 80 | D | 0 |
| 42 | 45 | F | 2 | 1 | 24 | 1 | D | 80 | 2 | T12 | 2 | 12 | 100 | D** | 0 |
| 43 | 23 | M | 6 | 1 | 44 | 2 | A | 50 | 4 | T5 | NA | 12 | 58 | C | 2 |
| 44 | 20 | M | 3 | 1 | NA | 1 | C | 67 | 2 | L1 | NA | 12 | 100 | E | 2 |
| 45 | 53 | M | 15 | 2 | 113 | 2 | C | 70 | 2 | T12 | 2 | 12 | 80 | D | 1 |
| 46 | 26 | F | 18 | 2 | 60 | 2 | C | 62 | 4 | T3 | 2 | 12 | 75 | D | 1 |
| 47 | 26 | M | 1 | 2 | 33 | 2 | A | 50 | 2 | T4 | 2 | 12 | 60 | A | 0 |
| 48 | 30 | M | 23 | 2 | 54 | 2 | A | 50 | 4 | T11 | 2 | 12 | 56 | A | 0 |
| 49 | 24 | M | 3 | 1 | 54 | 1 | B | 50 | 2 | L1 | NA | 12 | 60 | C | 1 |
| 50 | 35 | F | 2.5 | 2 | 43 | 2 | D | 90 | 3 | L1 | 2 | 12 | 100 | E | 1 |
| 51 | 35 | F | 16 | 1 | 217 | 1 | A | 50 | 3 | L1 | 0 | 12 | 66 | A | 0 |
| 52 | NA | F | NA | NA | NA | 2 | A | 50 | 2 | T2 | NA | 12 | 53 | A | 0 |
| 53 | 29 | M | 11 | 1 | 68 | 1 | A | 50 | 3 | T8 | 0* | 12 | 58 | A | 0 |
| 54 | NA | M | NA | 2 | NA | 1 | A | 90 | 2 | T12 | NA | 12 | 98 | D | 3 |
| 55 | 47 | F | 1 | 1 | 95 | 1 | D | 94 | 3 | T12 | 0* | 12 | 100 | E | 1 |
| 56 | 31 | F | 9 | 1 | 137 | 2 | A | 50 | 3 | L1 | 2 | 12 | 55 | A | 0 |
| 57 | 25 | M | 1.25 | 2 | 63 | 1 | D | 80 | 2 | T8 | 0* | 12 | 95 | D | 0 |
| 58 | 35 | M | 1.25 | 2 | 85 | 1 | A | 77 | 2 | T12 | 2 | 12 | 90 | D | 3 |
| 59 | 46 | M | 1 | 2 | 60 | 1 | A | 50 | 4 | T9 | 2 | 12 | 55 | A | 0 |
| 60 | 26 | M | 6 | 1 | 95 | 2 | A | 50 | 3 | T12 | 2 | 12 | 53 | A | 0 |
| 61 | 20 | M | 2 | 2 | 54 | 1 | A | 50 | 2 | T11 | 0 | 12 | 60 | A | 0 |
| 62 | 19 | M | 3 | 2 | 60 | 1 | A | 62 | 2 | T12 | 2 | 12 | 79 | D | 3 |
| 63 | 25 | F | 5.5 | 2 | 150 | 1 | A | 74 | 2 | T12 | 2 | 12 | 100 | D** | 3 |
| 64 | 22 | M | 3 | 1 | 92 | 1 | A | 50 | 4 | T8 | 2 | 12 | 58 | A | 0 |
| 65 | NA | M | NA | 1 | NA | 1 | A | 50 | 4 | T4 | NA | 12 | 54 | A | 0 |
| 66 | 34 | M | 16 | 1 | 96 | 1 | A | 50 | 2 | L1 | 2 | 12 | 54 | A | 0 |
| 67 | 25 | M | 1.5 | 1 | 18 | 1 | A | 50 | 4 | T11 | 2 | 12 | 50 | A | 0 |
| 68 | 22 | M | 1.5 | 1 | 21 | 1 | A | 50 | 3 | T12 | 2 | 12 | 52 | A | 0 |
| 69 | 28 | M | NA | 1 | NA | 1 | D | 85 | NA | T12 | NA | 12 | 97 | D | 0 |
| 70 | 23 | M | 1 | 4 | 10 | 1 | D | 94 | 2 | T12 | 2 | 12 | 100 | E | 1 |
| 71 | 57 | M | 5 | 1 | 23 | 2 | D | 82 | 2 | T12 | 2 | 12 | 100 | E | 1 |
| 72 | 26 | M | 1.5 | 1 | 52 | 2 | A | 50 | 2 | L1 | 2 | 12 | 56 | A | 0 |
| 73 | 30 | M | 6 | 2 | 19 | 1 | A | 66 | 2 | T11 | 2 | 12 | 84 | D | 3 |

1. A to A (hrs.): Accident to Admission in hours.

2. Injury mechanism: Falls=1; Motor Vehicle Collision=2; Sport=3; other=4.

3. A to OR (hrs.): Accident to operation in hours.

4. Group allocation: Surgery <24=1; ≥24=2.

5. Adm-AIS: Admission-AIS (American Spinal Injury Association (ASIA) Impairment Scale)

6. Adm-AMS: Admission ASIA motor score.

7. Fracture type Morphology: A0=1; A3/4=2; B2/3=3; C=4.

8. Surgical procedures: 0*: Cord decompressed by spine fixation - No laminectomy/corpectomy; 1: Corpectomy; 2: Laminectomy; 3: Corpectomy + Laminectomy.

9. F/U: Follow-up months.

10. FU-AMS: Follow-up AMS.

11. FU-AIS: Follow-up AIS.

12. AIS-C: AIS grade Conversion. There are three cases with AMS of 100 (Numbers 18, 42, & 63), we considered them as D** not E, because they had sensory deficit or neurologic deficit at least for some degrees in the urinary/stool incontinence.
